# Comparison of ertapenem non-susceptibility with 2-mercaptopropionic acid phenotypic tests in predicting NDM-1 and IMP-1 production in clinical isolates of Escherichia coli

**DOI:** 10.22088/cjim.11.4.426

**Published:** 2020

**Authors:** Zahra Shahandeh, Narges Kalantrai, Farahnaz Sadighian

**Affiliations:** 1 Department of Laboratory Sciences, Faculty of Paramedical Sciences, Babol University of Medical Sciences, Babol, Iran; 2 Cellular and Molecular Biology Research Center, Health Research Institute, Babol University of Medical Sciences, Babol, Iran

**Keywords:** 2-MPA, E. coli, Ertapenem, blaNDM-1, blaIMP-1

## Abstract

**Background:**

A routine phenotypic test has not been recommended for the detection of metallo-β-lactamases (MBLs) producing *Enterobacteriaceae* species such as *Escherichia coli*. The current study was conducted to compare the 2-mercaptopropionic acid (2-MPA) phenotypic method and ertapenem non-susceptibility test with polymerase chain reaction in predicting the production of MBLs in clinical isolates of *E. coli*.

**Methods:**

Antimicrobial susceptibility test for beta-lactam antibiotics were performed by disk diffusion method. All isolates which showed inhibition zones of ≤ 22 mm for CAZ and ≤ 27 mm for CTX were considered potential MBLs producing isolates. The production of MBLs was confirmed using 2-MPA compound. Also, susceptibility to ertapenem was evaluated in all isolates. Conventional PCR was performed to detect *bla*IMP-1 and/or *bla*NDM-1 genes in all potential MBLs producing *E. coli* isolates.

**Results:**

Of 259, 138 (53.3%) isolates were potential MBLs producing bacteria. One hundred and fifteen out of 138 (83.3%) isolates were susceptible to ertapenem. MBLs production was confirmed in 75/138 (54.4%) isolates by 2-MPA phenotypic method. The *bla*NDM-1 or/and *bla*IMP-1 genes were found in 30/75(40%) and 39/115(33.9%) isolates which were confirmed by 2-MPA and were susceptible to ertapenem, respectively. The sensitivity of 2-MPA method and ertapenem non-susceptibility test compared with PCR were 65.2% and 15.2%, and the specificity was 52.1% versus 82.6%, respectively.

**Conclusion:**

This study demonstrated that the 2-MPA phenotypic method does not have acceptable sensitivity and specificity in comparison with PCR, but its results are more reliable for the detection of MBL producing *E. coli* isolates compared with non-susceptibility to ertapenem.

Carbapenem-resistant *Enterobacteriaceae* (CRE) is defined as bacteria resistant to ertapenem, doripenem, imipenem and meropenem, and have been reported in different regions of the world. They cause serious problems for the treatment of infectious diseases and are classified as one of three urgent concerns by the Centers for Disease Control (CDC)(1). Infections with such bacteria, particularly in health care settings, are often life-threatening (2, 3). Different mechanisms may contribute to carbapenem resistance including carbapenems hydrolysis and/or producing the other beta-lactamases such as AmpC with mutations in porins at the outer membrane of gram negative bacteria.

Carbapenemase-producing *Enterobacteriaceae *(CPE) hydrolyze carbapenems by obtaining beta-lactamase genes through plasmids. These enzymes are categorized in three Ambler classes (4). The most prevalent identified carbapenemase in *Enterobacteriaceae* are KPCs (*klebsiella pneumoniae *carbapenemase), Metallo beta-lactamases (MBLs) including NDM, IMP, VIM and OXA-48-like in A, B and D classes, respectively. MBLs producing bacteria are often resistant to all beta-lactam antibiotics and bacteria which carry *bla*-NDM genes are the most prevalent carbapenemase producers in Asian countries (5). Several reports indicate that CPE, particularly those carrying various MBLs genes, distribute rapidly throughout the world (2, 6, 7).

Non-susceptibility to ertapenem is the most sensitive indicator for carbapenemase producing bacteria, as suggested by Clinical and Laboratory Standards Institute (CLSI). Based on our best knowledge, a routine standard phenotypic test for MBLs detection is not used in clinical laboratories. The modified Hodge test (MHT) for the identification of carbapenemase (KPC) in *Enterobacteriaceae* is recommended by CLSI. As the MHT test has low sensitivity and specificity for the detection of IMP, NDM and the other carbapenenemases, CLSI introduced different tests which are more sensitive and specific, although they are not recommended for routine clinical tests (8).

The efficacy of carbapenems have been documented in the treatment of some infection diseases caused by gram negative bacteria such nosocomial pneumonias. However, it is unclear that this effect is confirmed by all of published studies (9). For example a study which used CLSI and European Committee on Antimicrobial Susceptibility Testing (EUCAST) protocols on susceptibility to carbapenems showed that about 50% of CPE isolates which are susceptible to carbapenems (MIC assay) did not respond to treatment (10). Moreover, Adler et al. showed that MIC measurements alone may not be adequate in predicting the therapeutic efficacy of carbapenems against CPE. The complex interplay of different factors affect the resistant phenotype which is not identified by routine antimicrobial susceptibility test (AST) testing, therefore it is required to augment with a phenotypic test in order to detect CPE isolates (11).

New phenotypic methods have been developed using different compounds which inhibit metallo-betalactamase activity in bacteria such as 2-mercaptopropionic acid (2-MPA) and ethylenediaminetetraacetic acid (EDTA). Several studies have utilized different compounds and demonstrated that 2-MPA yielded more accurate results than other compounds (12-13). The current study was conducted to compare the 2-mercaptopropionic acid (2-MPA) phenotypic method and ertapenem non-susceptibility test with polymerase chain reaction (PCR) in predicting the production of MBLs in clinical isolates of *E. coli* as the most common infectious disease among *Enterobacteriaceae* throughout the world. 

## Methods

**Bacterial isolates:** Two-hundred fifty- nine non-duplicate *E. coli* isolates were obtained from various clinical specimens of patients admitted to three Babol university of Medical Sciences affiliated hospitals at Babol located in the North of Iran, between June 2015 and November 2016. Morphological features of bacterial colony, gram stain, and biochemical tests were used to identify the isolates as *E. coli*. 

**Antimicrobial susceptibility test (AST):** Disc diffusion assay was carried out on Mueller Hinton agar (MHA) for beta-lactam antibiotics including cefotaxime (CTX), ceftazidime (CAZ), cefpodoxime (CPD), cefepime (CPM), ertapenem (ETP), aztreonam (ATM) (Mast discs, UK) according to CLSI standard protocol (8).

**Phenotypic test for MBLs:** All isolates which showed inhibition zones of ≤ 22 mm for CAZ and ≤ 27 mm for CTX in the preliminary AST were considered potential MBLs producing *E. coli* (screening test). These isolates; I, were examined by 2 MPA and CAZ discs to confirm MBLs production (confirmatory test) (14, 15) and II, were evaluated for non-susceptibility to ertapenem (resistant or intermediate) and to at least one antibiotic from third-generation cephalosporins according to CLSI, 2016 protocol (8).

**Molecular analysis:** All potential MBLs isolates obtained by screening test were further assessed by PCR as the gold standard test (16).

**DNA extraction and PCR:** Total DNA was extracted from the bacterial suspension in sterilized normal saline using a commercial DNA extraction kit (Cinnagen Co., Iran) according to manufacturer's instruction. Then, PCR analysis of *bla*IMP-1 and* bla*NDM-1 genes was performed using a specific paired primer as follows: NDM-1, 5^ʹ^ACCGCCTGGACCGATGACCA3ʹ (forward) and 5ʹGCCAAAGTTGGGCGCGGTTG3ʹ (reverse); IMP-1, 5^ʹ^ TTGACACTCCATTTACAG3ʹ(forward) and 5^ʹ^ GATTGAGAATTAAGCCACTCT 3ʹ (reverse) (17). *Acinetobacter baumannii* and *E. coli* K12 were used as positive control strains for *bla*IMP-1 and* bla*NDM-1 genes, respectively (18, 19). Amplification was carried out as follows: initial denaturation at 94^o^C for 10 min; 30 cycles of 94^ o^C for 40s, 55^ o^C for 60s and 72^ o^C for 1min; and a final elongation step at 72^ o^C for 7min for the *bla*IMP-1 gene assay. The annealing temperature was optimal at 58^ o^C for the amplification of the *bla*NDM-1 gene. The PCR products were electrophoresed on a 2% agarose gel and evaluated by gel documentation system (Vilbert, Lourmat, France).

**Statistical analysis:** Data were analyzed by SPSS (ver.22) using descriptive statistics. The sensitivity and specificity of 2-MPA phenotypic and ertapenem non-susceptibility tests compared with PCR were evaluated. 

**Ethical consideration: **This study was permitted by the Ethics Committee of Babol University of Medical Sciences, Babol, Iran (Ethical number: MUBABOL.REC.1394.223).

## Results

**Figure 1:**
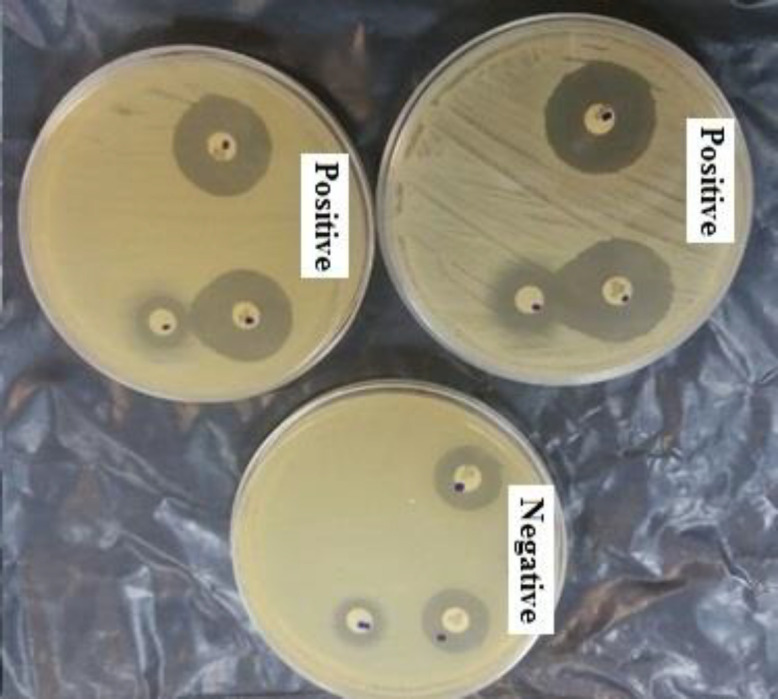
Inhibitory effects of 2-mercaptopropionic acid (2-MPA) on MBLs and non–MBLs producing E.coli. A distinct growth-inhibitory zone appeared between the disk containing CAZ and the filter disk containing 2-MPA in MBLs producing E.coli (positive). No change was seen around the two disks containing CAZ and the filter disk with 2-MPA in non-MBLs producers (negative)

One-hundred thirty-eight out of 259 (53.3%) isolates were identified as potential MBLs producing bacteria by the screening test (had inhibition zones of ≤ 22 mm for CAZ and ≤ 27 mm for CTX). MBLs production was confirmed in 54.4% (75/138) isolates by the confirmatory phenotypic test (2-MPA) (figure 1). Out of 138 potential MBLs, 23(16.7%) isolates were non-susceptible to ertapenem and resistant to one of third-generation of cephalosporins (CPD, CAZ and CTX) and 115(83.3%) isolates were susceptible to ertapenem. The *bla*NDM-1 and/or *bla*IMP-1 genes were found in 33.3% (46 /138) and 40% (30/75) isolated *E. coli* obtained by initial screening and confirmatory tests, respectively. The abovementioned genes were found in 25.4% (16.63) isolates despite negative confirmatory 2-MPA test. Among 75 MBLs producing bacteria confirmed by 2-MPA, 66(88%) isolates were susceptible to ertapenem and 27 out of 66 (40.9%) bacteria carried *bla*NDM and/or *bla*IMP genes (table 1). PCR analysis found that 7 out of 23 (30.4%) and 39 of 115(33.9%) isolates carried *bla*NDM-1 or/and *bla*IMP-1 genes (table 1).

The sensitivity and specificity of the abovementioned tests for the detection of MBLs compared with PCR are shown in table 2. The sensitivity of 2-MPA method and ertapenem non-susceptibility test compared with PCR were 65.2% and 15.2%, and the specificity was 52.1% versus 82.6%, respectively.

**Table 1:** The pattern of susceptibility to ertapenem and aztereonam, and the existence of blaNDM and blaIMP genes in potential MBLs producing E. coli isolates

C-2MPA*	Total	Susceptible ETP ATM N(%) N(%)	*bla*NDM&IMP genes+ ETP ATM N(%) N(%)	Non-Susceptible ETP ATM N(%) N(%)	*bla*NDM&IMP genes+ ETP ATM N(%) N(%)
**Positive**	75	66(88) 5(6.7)	27(40.9) 1(20)	9(12) 70(93.3)	3(33.3) 29(41.4)
**Negative**	63	49(77.8) 2(3.2)	12(24.5) 1(50)	14(22.2) 61(96.8)	4(28.6) 15(24.6)
**Total**	138	115(83.3) 7(5.1)	39(33.9) 2(28.6)	23(16.6) 131(94.9)	7(30.4) 44(33.6)

* Represent C-2MPA=Confirmatory test using 2-Mercaptopropionic acid; MBL=Metallobetalactamase; ETP=Ertapenem; ATM=Aztereonam

**Table 2:** Comparison of non-susceptibility to Ertapenem and confirmatory test by 2- Mercaptopropionic acid with PCR

	PCR POS.	PCR Neg.	Total
**Non-susceptible. Pos.**	7	16	23
**Non-susceptible. Neg.**	39	76	115
**Total**	46	92	138
**C-2MPA. Pos.**	30	45	75
**C-2MPA.Neg.**	16	47	63
**Total**	46	92	138

**Table 3:** Diagnostic accuracy indices of these phenotypic methods compared with PCR

	Sensitivity	Specificity	OR	NPV	PPV
**Non-susceptible to ertapenem**	15.2	82.6%	0.85	66	30
**2-MPA**	65.2%	52.1%	1.96	75	40

## Discussion

MBLs producing bacteria cause serious problems for the treatment of infectious diseases as they carry the most prevalent transferable carbapenemases which often cause resistance to all beta-lactam antibiotics (1). Generally, the genes responsible for the MBLs production are a part of an integron structure and carried on transferable plasmids or the genome (13). Since, a diverse number of genetic elements responsible for carbapenemase production are present in the bacterial genome, there is no standardized phenotypic test for the detection of MBLs producing bacteria in routine clinical laboratories (16). 

Non-susceptibility to ertapenem is the most sensitive indicator for carbapenemase producing bacteria (8). In this study, the sensitivity of non-susceptibility test to ertapenem in comparison to PCR analysis was very low (15.2%) while the specificity of this test was acceptable (82.6%). Also, the 2-MPA phenotypic method had moderate sensitivity and specificity but it was a more sensitive technique in detecting MBLs producing *E. coli* (DOR=1.96) in comparison with non-susceptibility to ertapenem (DOR=0.85) (table 2). The specificity and sensitivity of the 2-MPA phenotypic method for the detection of IMP enzyme were reported as 100% and 51.1% in *K. pneumoniae*, *E. cloacae* and *C. freundii* by Yan et al. (20). Also, Dobrzaniecka et al. reported that 2-MPA was more effective than EDTA compounds for the detection of MBLs, particularly in *Enterobacteriaceae *(12). Furthermore, the sensitivity and specificity of automated Microscan Walkaway system compared with non-susceptibility to ertapenem for the detection of IMP and NDM enzymes were 98% and 13%, respectively (21). A possible explanation for these differences obtained in our study and the aforementioned reports may be geographical variation, type of carbapenemase producing genes, bacterial species and sample size. Other findings obtained in the current study showed that 30.4% (7/23) of *E.coli* isolates diagnosed as potential MBLs based on non-susceptibility to ertapenem (8) carried *bla*NDM-1 and/or *bla*IMP-1 genes (table 2). A possible explanation for the lack of amplification of these genes in 69.6% (16/23) of the isolates may be due to existence of other carbapenemase producing genes or the presence of other resistance mechanisms. It is noteworthy that *bla*NDM-1 and/or *bla*IMP-1 genes were detected in 33.9% (39/115) of *E.coli* isolates which were susceptible to ertapenem. This finding is supported by Cetinkol et al., who reported that in spite of a low resistance rate to ETP (2.1%), 22.1% of *E.coli* isolates carried the *bla*NDM gene (22). Another study also reported that 18 (2%) isolates which were susceptible to ertapenem or other penems, carried carbapenemase producing genes such as *bla*NDM and *bla*IMP (21). Moreover, the existence of these genes in isolates susceptible to ertapenem and imipenem were reported by Karlowsky et al., 2017. Although the presence of carbapenemase producing genes in a low-number of such isolates has been reported, it can cause serious problems in the treatment of infectious diseases. Since penem susceptible isolates carry carbapenemase producing genes may act as reservoirs and can transfer these genes to other pathogens (5).

Aztreonam is a monobactam antibiotic that is not hydrolyzed by MBLs and is active against most strains of gram-negative microorganisms. The co-existence of MBL genes with other beta-lactamase genes including extended-spectrum beta-lactamases (ESBL) on a plasmid breaks down the aztreonam antibiotic (23). The existence of *bla*CTX-M, *bla*TEM and *bla*SHV genes were evaluated in these bacteria and our results showed that 100% of isolated *E. coli *that were non-susceptible to aztreonam which carried MBL genes, also carried the abovementioned genes. These findings are supported by other studies which demonstrate that 56.8% of isolated *E. coli* were resistant to aztreonam and also carried ESBL producing genes (24). Furthermore, a study performed in South Korea showed that 73.3% of NDM-producing *E. coli* was resistant to aztreonam (25). The limitations of our study are listed as follows: (i) antibiotic susceptibility was assessed only by disc diffusion method; (ii) AST was not performed for imipenem and meropenem; (iii) Only* bla*NDM and *bla*IMP genes were evaluated for MBLs production.

In conclusion, this study demonstrated that although the 2-MPA phenotypic method does not have acceptable sensitivity and specificity in comparison with PCR, its results are more reliable for the detection MBL producing *E. coli* isolates compared with non-susceptibility to ertapenem. The current work also recommends that *E. coli* isolates susceptible to ertapenem should be further evaluated for carbapenemase production by the other phenotypic tests. 
